# Squamous Cell Carcinoma of Unknown Primary: Multiple Skeletal Metastases Without Detectable Visceral Lesions

**DOI:** 10.7759/cureus.16525

**Published:** 2021-07-20

**Authors:** Kodama Tsukiji, Toshio Suzuki, Ryosuke Hashimoto, Masayuki Noguchi, Ikuo Sekine

**Affiliations:** 1 College of Medicine, School of Medicine and Health Science, University of Tsukuba, Tsukuba, JPN; 2 Medical Oncology, Faculty of Medicine, University of Tsukuba, Tsukuba, JPN; 3 Diagnostic Pathology, Faculty of Medicine, University of Tsukuba, Tsukuba, JPN

**Keywords:** squamous cell carcinoma of unknown primary, carcinoma of unknown primary, skeletal metastasis, diagnosis, differential diagnostic process

## Abstract

The appropriate diagnosis of carcinoma of unknown primary can be challenging for physicians, especially when they cannot apply a logical and ordered diagnostic process due to the unorthodox clinical characteristics. Here, we report the case of a 75-year-old woman presenting with multiple skeletal metastases with neither detectable visceral lesions nor site-specific distribution. Histological examination of a skeletal biopsy specimen unexpectedly revealed squamous cell carcinoma (SCC). Further specialized investigations led to the diagnosis of SCC of unknown primary. This case highlights the difficulty of the clinical differential diagnostic process in the quest for the occult primary tumor site.

## Introduction

Malignancy of undefined primary origin (MUO) is defined as metastatic malignancy without an obvious primary site identified after a limited number of radiological investigations [1]. A site-specific cancer is identified in approximately two-thirds of MUO cases following further specialized investigations [2,3]. The remaining cases are diagnosed as cancer of unknown primary (CUP), which is defined as metastatic cancer identified on the basis of final pathological histology, with no primary site detected despite further specialized investigations and review of the clinical and imaging data available [1].

The accurate diagnosis of CUP requires a conscientious effort in the search for the occult primary tumor site. The diagnostic approach for the primary site can be directed by considering the metastatic distribution of the tumor. However, in some cases, when the metastatic distribution is not site-specific, physicians cannot apply this strategy. To minimize the risk of delayed primary site-specific treatment of patients, physicians are required to move forward to the histological diagnosis after a limited initial screening. The pathological histology may reveal an unusual histological appearance for the sample site, adding to the diagnostic challenge.

Here, we describe a case of MUO presenting with multiple skeletal metastases. The histological examination of a skeletal biopsy specimen unexpectedly revealed squamous cell carcinoma (SCC). Further specialized investigations led to the diagnosis of squamous cell carcinoma of unknown primary (SCCUP). This case highlights the difficulty of the clinical differential diagnostic process.

## Case presentation

**Figure 1:**
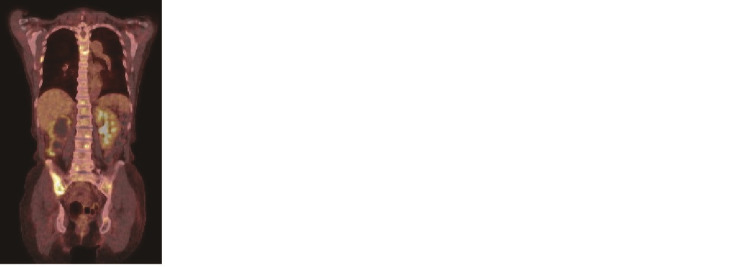
Initial PET-CT imaging obtained before the SCCUP diagnosis Coronal view of PET-CT showing multiple bone metastases through the entire body. PET-CT, positron emission tomography computed tomography; SCCUP, squamous cell carcinoma of unknown primary.

A 75-year-old Japanese woman presented with slowly progressing right buttock pain over two years. The patient did not have any clinical abnormalities except for tenderness in the right coxal bone. Due to elevated serum alkaline phosphatase (778 U/L, normal range: 104-338 U/L) and lactate dehydrogenase (279 U/L, normal range: 106-211 U/L) levels, bone involvement was highly suspected. Computed tomography (CT) revealed an unspecific distribution of multiple osteolytic and osteoblastic lesions involving the spine, scapula, costal bones, pelvic bones, and femur. It also revealed a previously diagnosed right hydronephrosis and renal stones without revealing the primary tumor origin. Blood tumor markers examination revealed elevated carbohydrate antigen 19-9 (CA19-9; 295.2 U/mL, normal range: <37.0 U/mL) and carbohydrate antigen 15-3 (CA15-3; 27.9 U/mL, normal range: <19.1 U/mL) levels. Other tumor markers, including carcinoembryonic antigen, SCC, cytokeratin 19 fragment, pro-gastrin-releasing peptide, carbohydrate antigen 125, and National Cancer Center-stomach-439, were within the normal range. Subsequently, the patient underwent a positron emission tomography scan. However, no radiotracer-avid visceral lesions or lymph nodes were visualized (Figure *1*). Hence, the primary origin of the corresponding skeletal metastatic lesion remained unknown.

Additional clinical investigations were performed to identify the primary site. Gastroscopy and colonoscopy ruled out a gastrointestinal tract origin, and CA19-9 levels were normalized two months after the initial examination. Similarly, mammography, ultrasonography, and magnetic resonance imaging (MRI) of the breast failed to identify a potential breast primary lesion. In addition, the CA15-3 levels returned to the normal range a month later. Urological review, including repeated urine cytology, showed no evidence of primary tumor. Therefore, a diagnosis of MUO was tentatively made in this patient.

**Figure 2:**
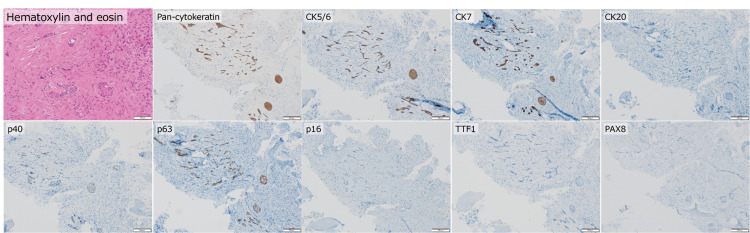
Histology of a biopsy sample from the ilium Several polygonal tumor cells with eosinophilic cytoplasm proliferate forming small nests and/or cord-like structures in the desmoplastic stroma. Immunohistochemically, the tumor cells were positive for pan-cytokeratin, CK5/6, CK7, p40, and p63, weakly positive for PAX8, and negative for CK20, p16, and TTF1  (scale bars: 100 µm for hematoxylin and eosin staining and 200 µm for other staining). CK, cytokeratin; PAX8, paired box 8; TTF-1, thyroid transcription factor-1.

After these initial examinations, a surgical biopsy was performed from the right ilium for the pathological investigation, and the patient was diagnosed with metastatic SCC. Immunohistochemically, the tumor cells were positive for pan-cytokeratin, p40, p63, cytokeratin (CK)5/6, CK7, weakly positive for paired box 8, and negative for estrogen receptor, progesterone receptor, CK20, gross cystic disease fluid protein-15, p16, and thyroid transcription factor-1 (Figure *2*). The immunohistochemical findings indicated that the tumor cells are from an epithelial origin (positive for pan-cytokeratin, CK5/6), with squamous cell characteristics (positive for p63 and p40). However, other findings were not conclusive to define the origin of this carcinoma.

Subsequent specialized investigations exploring the primary origin of the provisional SCCUP were performed. Colposcopy, transvaginal sonography, and cervical cytology test by a gynecologist did not reveal any primary lesion. A nasopharyngolaryngoscopy performed by an otolaryngologist did not show any evidence of primary lesion in the head and neck. Finally, we established a diagnosis of SCCUP presenting with multiple bone metastases exclusively.

The patient received a three-week regimen of carboplatin (area under the curve 6, day 1) plus paclitaxel (200 mg/m^2^, day 1) for four cycles, along with zoledronic acid administration. The tumor size was stable, and a reduced metastatic bone mineral density loss was observed after the chemotherapy cycles. Two months after the termination of the chemotherapy, new metastases were found in the cervical spine with progressive neck pain. The patient received palliative care, including palliative radiotherapy. Then, the patient was referred for hospice care and died nine months later (15 months after the initial treatment). Remarkably, the CT scans of the chest and abdomen during hospice care still did not disclose the primary lesion.

## Discussion

The skeleton is a relatively rare anatomic subsite of metastases in CUP. Brewster et al. reported that the liver was the most commonly recorded single site of metastases covering 28% of the cases, whereas skeleton accounted for only 5% of the cases [4]. Histological diagnosis of SCC is extremely rare in skeletal metastasis in CUP. Shih et al. evaluated patients diagnosed with skeletal metastases from an occult origin even after extensive investigations. They reported that the histological type was either adenocarcinoma (67%), poorly differentiated carcinoma (25%), or epidermoid carcinoma (8%) [5]. Indeed, in the current case, our top initial differential diagnoses included adenocarcinoma, lymphoma, multiple myeloma, and Ewing sarcoma, but SCC was unexpected.

We found two cases of SCC localized to the sternum in the literature [6,7]. These patients had a history of sternotomy incision for coronary artery bypass graft (CABG), and their carcinomas appeared to spread from the scar skin lesion. Because SCC that arises from the sternotomy scar skin after CABG had also been reported [8], these two cases represent the manifestation of skin cancer invasion. Thus, to the best of our knowledge, our case is the first to describe SCCUP with skeletal lesions alone.

Physicians usually investigate the origin of MUO with skeletal metastasis based on the information of the tumor prevalence in a specific geographical area and osteophilic property of various tumors [5]. However, we could not apply this rational approach for the following reasons. First, the metastatic sites of CUP with bone involvement are usually not limited to the skeleton alone but are more systemic with lymph node and visceral metastases [9-11]. Skeletal lesions are thought to be a late presentation of carcinomas. Therefore, physicians can usually access information regarding the geographical tumor distribution area of MUO. Second, we could not find any involvement of osteophilic tumors. Carcinomas in the breast, prostate, and lung generate skeletal metastases, whereas skeletal metastases of unknown origin usually are found to have roots in the lung or kidney after autopsy [12,13]. Our repeated comprehensive investigation through the course did not reveal any involvement of these organs. Furthermore, we attempted to perform a histological confirmation of SCC. Because we failed to find the primary site after reviewing the SCC-generating primary sites such as the head and neck, lung, esophagus, anus, uterine cervix, and skin, we decided to term the site of origin unknown in this case.

The etiology of this case is a matter of debate. One theory would be the common hypothesis for the etiology of CUP; the immune system might eliminate the primary SCC, and the metastatic sites alone survived and progressed. Comprehensive genetic profiling from the specimen would have solved this clinical question, although next-generation sequencing analyses of skeletal specimen need decalcification procedures leading to the DNA degradation.

## Conclusions

In conclusion, we reported a case of SCCUP with multiple skeletal metastases alone. Although SCCUP usually involves lymph node metastases, the unique metastatic distribution and unexpected histological result provoked our hesitation from applying the CUP-diagnostic framework. This case highlights the essence of internal medicine in difficulty of the clinical differential diagnostic process.
